# Frequency and Severity of Obsessive-Compulsive Symptoms/Disorders, Violence and Suicidal in Schizophrenic Patients

**Published:** 2012-06-30

**Authors:** S H Hosseini, M Zarghami, S Moudi, A R Mohammadpour

**Affiliations:** 1 Department of Psychiatry, Psychiatry and Behavioral Sciences Research Center, Mazandaran University of Medical Sciences, Mazandaran, Sari, Iran; 2 Department of Psychiatry, Babol University of Medical Sciences, Babol, Iran; 3 Department of Biostatistics, Mazandaran University of Medical Sciences, Mazandaran, Sari, Iran

**Keywords:** Compulsion, Obsession, Schizophrenia, Suicide, Violence

## Abstract

**Background:**

This study determined the prevalence and severity of obsessive-compulsive symptoms/disorder (OCS/OCD), aggression and suicidal in schizophrenic patients. Also we compared the prevalence and severity of aggression and suicidal in schizophrenic patients with and without OCS/OCD considering anxiety, depression and substance abuse as confounding factors.

**Methods:**

During 2007 and 2008, 100 schizophrenic patients were evaluated with Yale-Brown Obsessive Compulsive Scale, Positive and Negative Syndrome Scale, Beck Depression Inventory, Spilberger State/Trait Anxiety Inventory, Beck Scale for suicide Ideation, and Overt Aggression Scale.

**Results:**

OCS/OCD and suicidal attempts were seen in 33%, 10% and 12% of patients respectively. The most common form of aggression was against others (55%), and aggressive obsessions were seen in 10% of the patients. Comparing patients with and without OCS/OCD, there were no significant differences in the severity of schizophrenia, suicidal and overt aggression. The severity of overt aggression was related to the patients' age and education reversely. Also, there was a relationship between their suicidal thoughts and residence in the cities.

**Conclusions:**

High rate of aggressive obsessions and lack of relationship between severity of aggression and presence of OCD indicated that these patients did not act on these thoughts. The risk of suicide was more serious in patients living in the cities, and risk of violence was more serious in younger and less educated patients.

## Introduction

The annual incidence of schizophrenia ranges from 0.005 to 0.05 percent, and the lifetime prevalence is about 1 percent.[1] On the other hand; obsessive-compulsive disorder (OCD) which is a type of anxiety disorder is the fourth most common psychiatric diagnosis with a life time prevalence of 2 to 3 percent.[1] Obsessions and compulsions are common phenomena in schizophrenia, and subcorticofrontal pathway damage has been reported both in schizophrenia and OCD.[2] In multiple studies, the prevalence of obsessive compulsive symptoms (OCS) and OCD in patients with schizophrenia have been reported from 7.8 to 64 percent and from 2 to 36 percent respectively.[3][4][5][6][7][8][9][10][11][12][13] Some of these differences can be explained with regard to different diagnostic criteria, assessment methods, duration of schizophrenia, difficulty in differentiating OCS from schizophrenia symptoms and from probable side effects of atypical antipsychotics.[6][10][13] In the past, the presence of OCS was considered to be an indicator of favorable prognosis of schizophrenia.[7][9] But later, it was seen that these patients have more problems with performance, more social isolation and more resistance to treatment, compared with patients without OCS.[11]

Violence, suicide and substance abuse are the other common phenomena in schizophrenia.[1] Schizophrenic patients may be easily excited and have poor impulse control. Suicidal attempts, aggressiveness and also homicidal behavior are the common impulsive actions in schizophrenia. Suicide is the only cause of premature death in schizophrenia. Twenty to fifty percent of schizophrenic patients may attempt suicide and 10 to 13 percent of them may die because of suicide.[1] It is worth to mention that all of these phenomena (schizophrenia,[14][15] OCD,[16][17][18] violence,[19][20] suicide[21][22] and substance abuse[23][24] have an important common etiologic factor; serotonin.

Regarding the different rates of the above phenomena and their common biological etiology, this study was designed to determine the prevalence and clinical severity of OCS/OCD, aggression and suicidal in schizophrenic patients. Also we compared the prevalence and clinical severity of aggression and suicidal in schizophrenic patients with and without OCD/OCS considering anxiety, depression and substance abuse as confounding factors.

## Materials and Methods

In a cross-sectional study with simple sampling during 2007 and 2008, 100 patients admitted to Zare Teaching Hospital of Sari (Iran) with the diagnosis of schizophrenia based on Diagnostic and Statistical Manual of Mental Disorder Fourth Edition Text Revision (DSM-IV-TR)[1] criteria were studied after obtaining the written informed consent from the patients and their guardians. Cases with substance dependency, endocrine and neurologic diseases, and mental retardation were excluded. For at least one week, all of the patients did not use antipsychotic drugs and were studied in the first ten days of hospitalization. In each patient, age, sex, age of schizophrenia onset, duration of symptoms of OCD and schizophrenia, level of education, marital status, type of schizophrenia based on DSM-IV-TR criteria, family history of psychiatric disorders, drug history and past psychiatric ward admission were recorded. OCS was evaluated by Yale-Brown Obsessive Compulsive Scale (Y/BOCS).[25] In the presence of OCD documented with the structured clinical interview (SCID-I)[1][25] based on DSM-IV-TR,[1] its severity was assessed by Y/BOCS. Severity of schizophrenia was assessed by Positive and Negative Syndrome Scale (PANSS).[25] The history of suicidal ideas and attempts and aggression were recorded according to the reports of the patient and his/her family members and, in the case of suicidal attempt in recent hospitalization, the severity of suicidal ideation was evaluated using Beck Scale for suicide Ideation (BSS).[25] Depression and anxiety in each patient were measured with Beck Depression Inventory (BDI),[25] and Spilberger State/Trait Anxiety Inventory (STAI)[26][27] respectively. Aggression intensity in recent hospitalization and during the whole period of the disorder was assessed by Overt Aggression Scale (OAS).[28][29][30] The study was approved by the Research Council of the Research Center for Psychiatry and Behavioral Sciences and the Ethics Committee of Mazandaran University of Medical Sciences and Health Services.

The Diagnostic and Statistical Manual of Mental Disorders (DSM) is published by the American Psychiatry Association to provide a common language and standard criteria for the classification of mental disorders. A "text revision" of the DSM-IV (DSM-IV-TR), was published in 2000.[1]

The structured clinical interview (SCID-I) is a clinician-administered, semi-structured diagnostic tool to determine major (DSM-IV Axis I disorders). The overall reliability was fair to good in patient samples (weighted kappa=0.61). Evaluating the validity of SCID-I, the positive predictive value was high, but the negative predictive value was low.[25]

The Yale/Brown Obsessive Compulsive Scale (Y/BOCS) is the gold standard for identification of the content of OCS. This semi-structured interview has acceptable internal consistency; Cronbach’s alphas ranged from 0.51 to 0.91 for the two subscales and the total scale. The total score has demonstrated excellent joint reliability and test-retest reliability indicated good stability; intra-class correlation coefficients (ICCs) of 0.80-0.99 and 0.81-0.97 respectively. The scale demonstrated moderate convergence with questionnaire measures of OCS (r=0.33-0.62).[25]

The Positive and Negative Syndrome Scale (PANSS) is a scale used for measuring severity of psychopathology in patients with schizophrenia. The 30 items from the PANSS are summed to determine scores on Positive, Negative, and General Psychopathology subscales and total PANSS score. The scale has well to excellent joint reliability (ICCs above 0.80) and internal consistency (Cronbach’s alpha of 0.73-0.87). Studies comparing subscales of the PANSS with similar scales have consistently demonstrated good concurrent validity.[25]

The Beck Scale for suicide Ideation (BSS) is a valuable tool for clinicians to examine suicidal intent in patients. Cronbach’s alpha (internal consistency) and joint reliability for the BSS was 0.89-0.96 and 0.83 respectively. BSS was significantly correlated with the Beck’s scales for depression and hopelessness.[25]

The Beck Depression Inventory (BDI) is a test to measure the severity and depth of depression which shows high internal consistency (Cronbach’s alpha ranged from 0.76 to 0.95). Internal consistency was an alpha value of 0.86, and test-retest reliability showed r=0.81-0.83. Correlation between the BDI and other standard measures of depression severity was 0.72.[25]

The Spilberger State/Trait Anxiety Inventory (STAI) is an instrument for measuring anxiety. This inventory differentiates between two kinds of anxiety. The brief short lived temporary condition of "state anxiety" is characterized by subjective, consciously feelings of worry, tension, nervousness, apprehension and increased autonomic nervous system activity. This form of anxiety fluctuates over time and varies in intensity. The more general and extended long-standing "trait anxiety" is relatively stable and indicates individual differences in anxiety proneness and a general tendency to respond with apprehension to perceived threats in the environment. The test-retest reliability coefficients decreased for the trait-anxiety scale ranged from 0.65 to 0.86, whereas the range for the state-anxiety scale was 0.16 to 0.62. Correlations between STAI and other measures of trait-anxiety were from 0.52 to 0.80.[27][31]

The Overt Aggression Scale (OAS) is a valid instrument which was designed to assess various manifestations of aggressive behavior. On the OAS, aggression is divided into four categories: verbal aggression, physical aggression against objects, physical aggression against self, and physical aggression against others.[28][29] Three inter-rate reliability trials are reported, with infraclass correlation coefficients of 0.7 for aggression and 0.87 for most restrictive intervention.[30]

Results were expressed with descriptive statistics as mean±standard deviation (SD) for continuous variables, unless otherwise stated. The differences between groups were analyzed using Student's unpaired t-test or Χ^2^ test, when appropriate .T-test was used for quantitative variables (e.g.; age, positive, negative and general symptoms scores) and X^2^ test was used for qualitative variables (e.g.; Marriage status, living location, suicidal ideas). Analysis for each group questionnaire’s data of the Overt Aggression Scale (OAS) was done by Analysis Of Variance (ANOVA). All statistical analyses were achieved using SPSS software (SPSS version 11.5, Chicago, IL, USA). For all tests, significance was defined as p<0.05.

## Results

Demographic and clinical characteristics of patients with schizophrenia based on presence/absence of OCS/OCD are presented in Table 1. Ranks of hospitalization were 3±2, and most of them were in the first and the second admission (each 28%). Two patients had obsessive symptoms, 7 of them had compulsive symptoms and 24 patients had both obsessions and compulsions.

**Table 1:** Demographic and clinical characteristics of schizophrenic patients based on presence/absence of OCS/OCD.

**Schizophrenic patients**		**Without OCS^a^**	**With OCS/without OCD**	**With OCDb**	**Total**
Sex	Female	17	7	2	26
	Male	50	16	8	74
Age (year)		34.97±9.18	35.13±9.74	34.60±10.94	34.9±9.3
Marriage (%)	Single	38	16	8	62
	Married	12	4	1	17
	Divorced	17	3	1	21
Living location(%)	Urban	46	16	10	72
	Rural	21	7	0	28
age of schizophrenia onset (years old)		22.91±7.49	24.39±8.44	21.70±7.28	23.1±7.6
Duration of schizophrenia (years)		11.9±7/71	10.3±7.45	12.8±8.25	11.6±7.6
Positive symptoms score (PANSS)		23.42±6.42	23.48±5.29	24.80±4.84	23.57±6
Negative symptoms score (PANSS)		24.25±7.54	24.04±5.14	22.60±6.22	24.04±6.8
General psychopathology score (PANSS)		40.51±7.14	41.26±4.05	40.30±5.75	40.66±6.3
Supplemental score (PANSS)		6.77±3.16	8.69±3.38	8.3±2.40	8.05±2.91
Total score (PANSS)		95.01±14.61	97.30±9.23	95.40±12.00	95.58±13.23

^a^ OCS=Obsessive-Compulsive Symptoms

^b^ OCD=Obsessive-Compulsive Disorder

Based on the structured clinical interview (SCID-I) considering DSM-IV-TR criteria, 10 patients (10%) had OCD; 6 of them had OCD before the onset of schizophrenia. The most common symptoms of obsession were contamination obsessions (17%) and aggressive obsessions (10%). Sexual obsessions (4%), symmetry (3%), somatic (3%), morbid doubt (2%) and other obsessions (7%) had lower frequencies. The most common compulsion was washing compulsion (22%). Based on Y/BOCS, 3%, 6%, and 1% had moderate and severe OCD respectively. None of them had very severe OCD. Ninety one percent of the patients had history of receiving anti-psychotic medications more than 6 weeks before the recent hospitalization (69% classic anti-psychotics, 27% clozapine, 28% risperidone, 8% olanzapine and 11% two atypical anti-psychotics). Twenty eight percent of the patients had suicidal ideas and 15% had past history of suicidal attempts. Among the 15 patients, 11 patients had attempted suicide once. The most common method of suicide was drug consumption (7 cases). Other methods included hanging, self-poisoning, wrist cutting and self-burning. Among the 100 patients, suicidal thoughts and attempts were detected in 20 and 2 of them in their recent admission respectively. The intensity of their action based on BSS was 10 and 14 respectively. There were no significant differences between the suicidal ideas in recent hospitalization and age, sex, education, marital status, ranks of hospitalization, severity of schizophrenia symptoms, depression and anxiety scores. But these ideas were higher in patients who were residents of urban areas (p=0.03). Only about 72 to 84 percent of the patients cooperated with the researchers answering Beck Depression Inventory (BDI) and Spilberger State/Trait Anxiety Inventory respectively. Anxiety and depression rates of the patients are shown in Table 2 and 3.

**Table 2:** State and trait anxiety in schizophrenic patients.

**Severe (%)**	**Relative severe (%) **	**More than moderate (%) **	**Less than moderate (%) **	**Mild (%)**	**Anxiety severity **
3	7	28	24	10	State anxiety
2	5	22	31	12	Trait anxiety

**Table 3:** Depression in schizophrenic patients.

**Depression severity**	**Mild (%)**	**Moderate (%)**	**Severe (%)**
****	28	12	0

According to OAS, overt aggression rates are shown in Table 4. There was significant negative relation between the severity of overt aggression and age and education (p<0.05) (Pearson correlation rate was near to-1).

**Table 4:** Overt aggression rates in schizophrenic patients.

**Categories of overt aggression**	**Verbal aggression (%)**	**Physical aggression against objects (%)**	**Physical aggression against self (%)**	**Physical aggression against others (%)**
Recently	23	11	2	55
Lifetime	18	8	3	69

Considering obsessions and compulsions, there was no significant relationship between demographic factors, ranks of hospitalization, duration of symptoms and severity of schizophrenia in schizophrenic patients with OCD, with OCS/without OCD, and without OCS (p>0.05) (Table 1). There was no significant relationship between OCS severity and schizophrenia. The relationship between the past/present suicidal thoughts/attempts and aggression severities, in the presence or absence of OCD were not significant. Likewise, there was no significant relationship between the consumption of atypical anti-psychotics before recent admission, and OCS. There were no significant relationships between the severity of schizophrenia and the presence or absence of OCS/OCD before onset of schizophrenia. No significant relationship was mentioned between flat affect and OCS/OCD. However, the relationship between the total scores of PANSS and OAS was significant (p=0.001).

## Discussion

In this study, the prevalence of OCS and OCD in schizophrenic patients was estimated to be 33% and 10% respectively, which are more prevalent than the general population.

These results are in accordance with Berman, Byerly, Kayahan, Poyurovsky and Haan studies.[2][5][7][11] As Byerly has reminded, in studies with broader screening tools, the presence of OCS and OCD were reported more.[4] On the other hand, the differences in the prevalence of symptoms in different studies can be due to characteristics of the disorder, type of admission of the patients, chronicity of schizophrenia, course of the disorder and type of consumed drugs.[3] The present study has shown higher rates of symptoms and disorder than another Iranian study which has used the same screening tools.[12] In this study, 6 out of 10 patients had OCD before the onset of schizophrenia; which is similar to Poyurovsky’s study.[11] The high rate of OCS and OCD triggers this tip in mind that these may be accompanied beyond a simple comorbidity.

Mean intensity of OCD based on Y/BOCS was 17.9±4.2. There was mild, moderate, and severe OCD in 3%, 6%, and 6% of patients respectively. But none of them had very severe OCD. Ghoreishi (2003) also had not found very severe obsessions and compulsions in Iranian schizophrenic patients.[12]

The most common obsession was contamination (17%), followed by aggressive (10%) and sexual (4%) obsessions; and the most common compulsion was washing (22%), followed by checking compulsion (17%). In Hann and colleagues' study, these obsessions were more prevalent, but checking, arranging, repetition and washing compulsions had higher prevalence respectively.[5] The high percentage of aggressive obsessions in the present study and lack of significant relationship between the severity of overt aggression and the presence or absence of OCD indicates that these patients do not act on their obsessive thoughts.

This study did not confirm other studies[6][7] and found no correlation between atypical anti-psychotic drugs consumption and OCD. Considering the low percent of patients whose symptoms of OCD were seen after the onset of schizophrenia and anti-psychotic drug consumption (4%), it can not be said whether initiation or exacerbation of OCD symptoms is due to drugs effect on serotonin. Longitudinal and interventional studies are needed to collect evidence for the relationship between drugs and symptoms of OCD.

There was no significant relationship between demographic factors, ranks of hospitalization, duration of symptoms, age and severity of schizophrenia in 3 groups of with OCD, with OCS/without OCD, and without OCS. Also, there was no relationship between severity of OCD symptoms and severity of schizophrenia. These results are in accordance with the studies of Poyurovsky and Byerly et al.,[4][11] but are in contradiction with Haan and Tibbo et al., who reported a significant relationship between OCD symptoms and reduction in negative schizophrenic symptoms,[5][9] and with Nechmad et al. who reported a positive significant relationship between negative symptoms (especially flat affect) and OCD symptoms.[32] The results of our study were also contradictory to Kayahan et al. study[3] which reported a correlation between severity of OCD with positive, general and total scores of the PANSS. It should be mentioned that Kayahan's study evaluated only the course of first episode of schizophrenia and in other studies, patients with simultaneous OCD symptoms and schizophrenia were more dysfunctional and re-sistant to treatment.[11][33]

Considering 20-50% rate of suicidal attempt in schizophrenic patients,[1] this figure is reasonable in our study; 28% lifetime suicidal ideas, 15% lifetime suicidal attempt and 20% suicidal ideas in recent admission. The most common method of suicidal attempt in this study was drug overdose, which is identical to other studies conducted in the field of suicide in Iran.[34] It can be justified with more access to drugs rather than weapons, which is the most common methods of suicide in Western countries.[31][35][36] Unlike Sevincok's study which showed more suicidal thoughts and attempts in patients with OCS,[37] Apter’s study showed the lower risk of suicidal attempts in patients with OCD.[38] There were no significant differences between schizophrenic patients with and without suicidal ideas and attempts regarding OCD/OCS in our study.

Also, there were no significant differences between the existence and absence of suicidal ideas in recent admission and age, gender, education, marital status, ranks of hospitalization, severity of schizophrenic, depression and anxiety symptoms, which is in accordance with the findings of Montross and Sevincok.[37][39] However, there was no relationship between the suicidal and gender in Reutors’s and Ghoreishi’s studies.[33][40] We found that suicidal ideas in urban patients are significantly more than in the rural ones. Suicidal thoughts in recent hospitalization did not differ significantly between schizophrenic patients with and without OCD/OCS, even after the elimination of the effects of depression and anxiety. Demographic factors, duration and severity of symptoms of schizophrenia and aggression were the same in patients with and without OCD/OCS in our study. Our findings that there was no significant relationship between suicidal ideas and severity of depression are different from two other studies.[34][39]

One of the strong points of this study was to consider the various confounding factors (including depression, anxiety and substance dependence) in suicidal and aggressive behavior as well as OCS. Another strong aspect was the exclusion of other diagnoses such as schizoaffective and schizophreniform disorder. However, the cross-sectional nature of the study and the recall bias of information sources were important limitations of our research. We suggest a longitudinal study in the future to evaluate the course of OCD/OCS and their effect in the treatment of schizophrenic patients.
